# Neurodegeneration with brain iron accumulation: A case report

**DOI:** 10.1590/S1980-5764-2016DN1002014

**Published:** 2016

**Authors:** Daniel Nassif, João Santos Pereira, Mariana Spitz, Cláudia Capitão, Alessandra Faria

**Affiliations:** 1 Movement Disorders Sector, Neurology Service, Pedro Ernesto University Hospital, State University of Rio de Janeiro, Rio de Janeiro RJ, Brazil.; 2 Post Graduate Stricto Sensu Program in Medical Sciences, School of Medical Sciences, State University of Rio de Janeiro, Rio de Janeiro RJ, Brazil.

**Keywords:** PKAN, NBIA, PANK2

## Abstract

Pantothenate kinase-associated neurodegeneration (PKAN) is an autosomal recessive disorder caused by mutation in the *PANK2* gene. It is characterized by abnormal brain iron accumulation, mainly in the globus pallidus. PKAN is included in a group of disorders known as neurodegeneration with brain iron accumulation (NBIA). We report a case of atypical PKAN with its most characteristic presentation, exhibiting marked psychiatric symptoms, speech disorder and focal dystonia. Brain MRI has great diagnostic importance in this group of disorders and, in this case, disclosed the eye-of-the-tiger sign. Genetic testing confirmed the diagnosis.

## RESUMO

Neurodegeneração associada à pantotenato-quinase (PKAN) é uma entidade autossômica recessiva causada pela mutação do gene *PANK2.* Caracteriza-se por depósito cerebral anormal de ferro, particularmente nos globos pálidos. PKAN faz parte de um grupo de desordens conhecidas como neurodegeneração com acúmulo cerebral de ferro (NBIA). Relatamos um caso de PKAN atípica com sua apresentação mais característica, sendo evidentes sintomas psiquiátricos marcados, distúrbio da fala e distonia focal. A ressonância magnética de crânio possui grande importância diagnóstica neste grupo de desordens, e neste caso, demonstrou o sinal do olho de tigre. O teste genético confirmou o diagnóstico.

## INTRODUCTION

Neurodegeneration with brain iron accumulation (NBIA) syndromes are neurodegenerative disorders whose main feature is the abnormal accumulation of iron, predominantly in the globus pallidus. Clinical findings are heterogeneous, but typically include progressive extrapyramidal disorder, with variable involvement of the pyramidal, peripheral nervous, autonomic, cognitive, psychiatric, visual and cerebellar systems.^1^ ^,^ ^2^

Pantothenate kinase-associated neurodegeneration (PKAN) represents the most prevalent form of NBIA, accounting for 50% of cases. This is caused by mutation in the *PANK2* gene.^3^ PKAN is subdivided into the classic form, with early onset and rapid evolution, and the atypical form, with later onset and slower progression.^4^

The use of brain magnetic resonance imaging to disclose the accumulation of brain iron is important for confirming the diagnosis and also facilitates differential diagnosis among NBIAs. In PKAN, the presence of the eye-of-the-tiger sign is a defining characteristic.^5^ Genetic testing is available for most of the NBIA-associated syndromes, including PKAN.^1^

Currently, however, only symptomatic treatment of these disorders is possbile.^1^ ^-^ ^3^

We report a case of atypical PKAN with typical radiological findings and genetic confirmation of the entity. 

## CASE REPORT

A 21-year-old female student presented with a four-year history of irritability, learning difficulties with decline in academic performance, and progressive emotional lability. The neurological examination revealed the presence of dysarthria, focal dystonia involving the left hand, and facial tics, as well as the presence of hyperreflexia and spasticity of the lower limbs, with flexor plantar reflex. Tests for strength, coordination and sensitivity were unremarkable. No parkinsonism, chorea or myoclonus was evident. The neuro-ophthalmologic exam, including fundoscopy was normal. The patient had 10 years of education and a total score of 23 on the Mini-Mental State Exam (MMSE). She scored a total of 39 points on the Beck Depression Inventory (BDI), indicating marked depressive symptoms. There was no history of neurological disease or consanguinity. 

The patient was submitted to subtests from the WAIS III battery (Wechsler Adult intelligence Scale - 3^rd^ edition) and to specific tests for assessing attention, memory, language, praxis, perception and executive functions, and proved cooperative and communicative during the exam. The results indicated a worse performance on processing speed, attention switching, long-term memory, verbal reasoning, abstraction, verbal fluency, planning and perceptual organization (including strategy in constructional praxis). Inhibitory control was mildly impaired whereas working memory was within normal limits but with fluctuating performance on specific tasks. The phonoaudiological assessment revealed mild dysarthria, with impairment of phonation and articulation, in addition to sporadic gagging.

**Figure 1:**
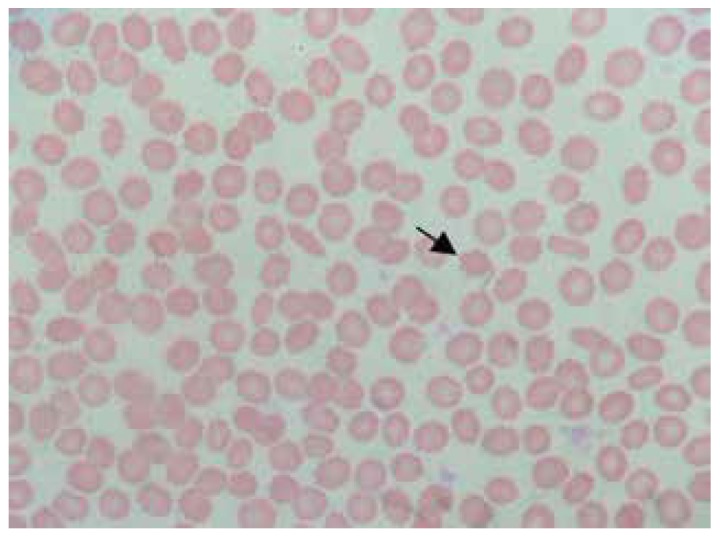
Blood smear shows numerous acanthocytes.

Laboratory tests, including full blood count, biochemical evaluation, renal and liver function tests were normal. Sera copper and ceruloplasmin concentrations, as well as urinary copper level, were also normal. Blood smear showed a large number of acanthocytes (Figure 1).

**Figure 2:**
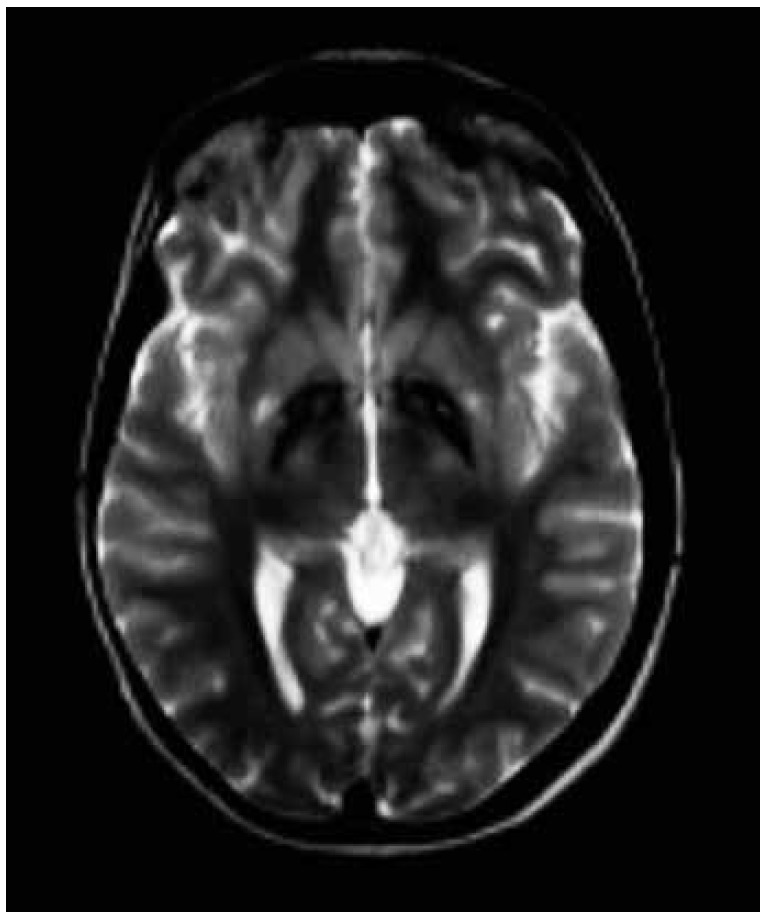
T2-weighted magnetic resonance imaging showing symmetrical central hyperintensity surrounded by hypointense signal in globus pallidus, giving "eye of tiger" appearance.

A T2 weighted brain magnetic resonance imaging demonstrated symmetrical central hyperintensity surrounded by hypointense signal in globus pallidus, consistent with the "eye-of-the-tiger" sign. T2* demonstrated low signal in corresponding areas from iron deposition (Figures 2 and ^3^).

**Figure 3:**
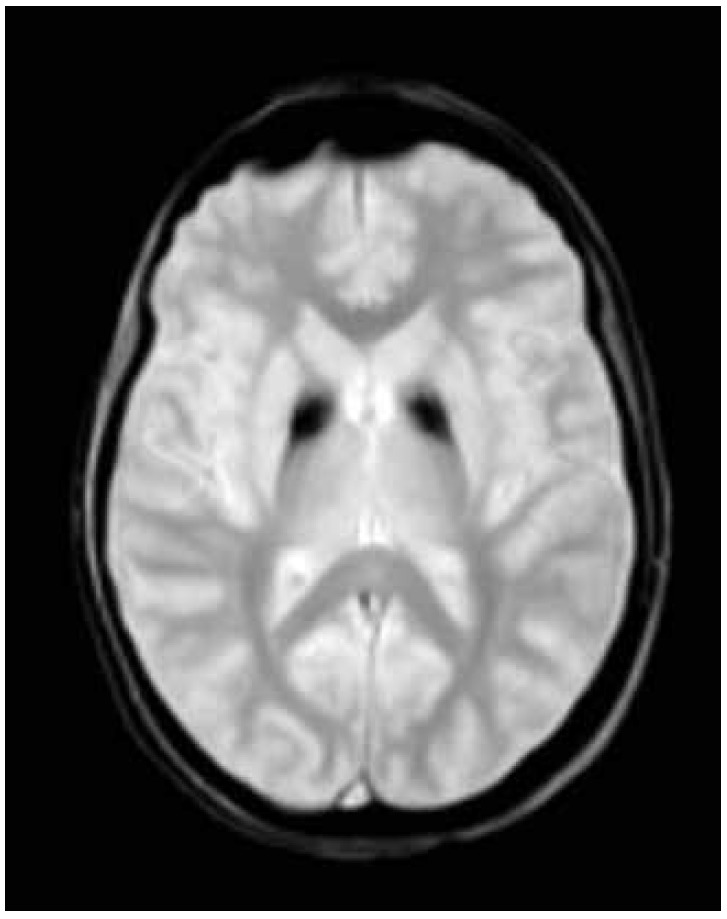
T2* shows low signal in corresponding areas from iron deposition.

Given the suspected diagnosis, genetic testing was performed and the molecular analysis of itron 5 of the *PANK2* gene revealed the presence of the c.1537-3C>G (IVS5-3C>G) heterozygous mutation. This variant located at the splicing site induces failure during the processing of the messenger RNA of the pantothenate-kinase protein, resulting in a non-functional protein. This finding confirms the diagnosis of pantothenate kinase-associated neurodegeneration (PKAN).

## DISCUSSION

Hallervorden-Spatz syndrome was first described in 1922 by two German neuropathologists, Julius Hallervorden and Hugo Spatz, whose studies were based on pathological specimens obtained under Nazi euthanasia programs in individuals with physical and intellectual disabilities.^6^ ^,^ ^7^ For decades, despite the clinical heterogeneity, the majority of patients with pathological or radiological evidence of brain iron accumulation were diagnosed as having Hallervorden-Spatz, although they probably had other NBIA syndromes.^8^

The first subtype of the Hallervorden-Spatz syndrome, identified by the mutation in the *PANK2* gene and specific radiological and clinical findings, was denominated pantothenate kinase-associated neurodegeneration or PKAN.^9^

In 2003, a new nomenclature for the syndrome was proposed prompted by the anti-ethical activities of the neuropathologists who originally described the syndrome and due to the recognition of genetic mutations associated with the clinical syndrome, particularly the *PANK2* gene. This led to introduction of the terms neurodegeneration with brain iron accumulation (NBIA) and pantothenate kinase-associated neurodegeneration (PKAN) to denote patients with suspected or proven mutation in the PANK2 gene.^4^ ^,^ ^8^ ^,^ ^10^

A number of genes associated with NBIA have been identified, including PANK2, PLA2G6, FA2H, C19orf12, ATP13A2, CP, and FTL. The terminology of the syndromes associated with these genes follows a system in which the first letters refer to the physiopathogenic molecular base involved and the last letters to "associated neurodegeneration", for example, pantothenate kinase-associated neurodegeneration or PKAN, as depicted in Table 1.^1^ ^,^ ^3^

**Table 1:** NBIA subtypes described to date with acronym and mode of inheritance.

Autosomal recessive
Pantothenate kinase-associated neurodegeneration (PKAN)
Phospholipase A2-associated neurodegeneration (PLAN)
Mitochondrial membrane protein-associated neurodegeneration (MPAN)
Fatty acid hydroxylase-associated neurodegeneration (FAHN)
Coenzyme A synthase protein-associated neurodegeneration (CoPAN)
Kufor-Rakeb Syndrome
Woodhouse-Sakati Syndrome
Aceruloplasminemia
Autosomal dominant
Neuroferritinopathy
X-linked dominant
Beta-propeller protein-associated neurodegeneration (BPAN)

NBIA: Neurodegeneration with brain iron accumulation.

PKAN accounts for 50% of NBIA cases.^3^ It is a rare autosomal recessive disorder associated with mutation in the PANK2 gene located on chromosome 20p13 and encodes pantothenate kinase, a key regulating enzyme of coenzyme-A synthesis.^1^ ^,^ ^11^

PKAN is subdivided into two main types, based on age of onset, symptoms and progression: (1) classic (2) atypical.^4^ Some cases have overlapping features^3^ and others do not fit this subdivision.^11^

The onset of classic PKAN generally occurs at around three years of age and before six years in 90% of cases. It typically presents with gait difficulties.^2^ ^,^ ^12^ Patients present pyramidal and extrapyramidal signs with marked dystonia.^2^ The dystonia generally dominates the clinical picture and begins assymetrically.^12^ Severe tongue protrusion dystonia can occur.^13^ The presence of dystonic opisthotonus or oromandibular dystonia suggests inclusion of PKAN as a differential diagnosis.^14^ The neuro-ophthalmologic exam can reveal pigmentary retinopathy and saccade and pupil abnormalities.^15^ Some patients have acanthocytes in peripheral blood.^16^

The progression of classic PKAN is not linear, exhibiting periods of marked worsening. Bulbar compromise can lead to nutritional and respiratory problems.^3^ Loss of walking ability occurs within 10-15 years of disease onset.^4^ Some patients evolve to death within the first decade of disease onset whilst others reach adulthood.^3^

Atypical PKAN is more heterogeneous than the classic form, emerging in the second or third decade of life with slower evolution.^3^ ^,^ ^4^ ^,^ ^8^ Psychiatric symptoms and speech disorders are characteristic,^11^ and were found in the present case. Psychiatric symptoms include depression, emotional lability, impulsivity, aggressivity and tourettism with motor and verbal tics.^17^ Speech disorders include dysarthria, hypophonia, spasmodic dysphonia and palilalia. Pigmentary retinopathy is rare.^3^ ^,^ ^4^

Unlike the classic form, motor involvement is less marked, with loss of walking at 15-40 years of disease.^4^ ^,^ ^17^ Dystonia tends to dominate the clinical picture in adolescents, while patients over 20 years of age tend to present parkinsonism as the predominant clinical symptom. Pyramidal signs can occur. Motor involvement may not be significant in the initial stage of the disease.^3^

Brain magnetic resonance imaging is an important diagnostic tool. The characteristic finding is the eye-of-the-tiger sign, characterized by bilateral hypointensity of the globus pallidus with a central area of hyperintensity on T2-weighted images. Hypointensity in substantia nigra may be seen in some patients. Areas of hypointensity at sites of iron deposits are visible on T2*.^18^ The area of hypointensity correlates pathologically with abnormal iron deposition, while central hyperintense signal occurs due to neuronal loss with gliosis.^19^

As the disease progresses, the area of hypointensity can dominate the radiological presentation. It is believed that the central hyperintense area can disappear.^8^ ^,^ ^18^ There are controversial reports in the literature on the correlation of mutation in the *PANK2* gene and the presence of the eye-of-the-tiger sign.^4^ ^,^ ^20^ This same sign has been described in other pathologies, such as neuroferritinopathy, corticobasal degeneration, multiple system atrophy^18^ and in a case report with syndromic diagnosis of early onset parkinsonism, with response to dopaminergic therapy.^21^

The management of PKAN remains symptomatic, as is the case for other NBIAs. Dystonia can be controlled with the use of benzodiazepines, anticholinergics and botulinum toxin. Baclofen can be used for relieving spasticity.^1^ ^,^ ^3^ Stereotactic surgical modalities such as thalamotomy^22^ and palidotomy^23^ and deep brain stimulation of the internal globus pallidus,^24^ can help control symptoms but do not arrest disease progression.^1^

Despite the few options, the benefit of using iron chelates, such as deferiprone, is questionable.^1^ ^,^ ^3^

It can be concluded that, although rare, neurodegeneration with brain iron accumulation syndromes should be included as part of the differential diagnosis in patients with progressive extrapyramidal syndrome, particularly when brain iron deposits are depicted on magnetic resonance imaging. Multidisciplinary rehabilitation programs can promote satisfactory conditions in these patients and contribute toward improving their quality of life.
